# Postoperative Sore Throat in the Postanesthetic Care Unit: Incidence, Contributing Factors, and Association With Airway Management

**DOI:** 10.7759/cureus.98473

**Published:** 2025-12-04

**Authors:** Inês Sousa Braga, Igor Ferreira, Andreia Sá, Humberto Machado

**Affiliations:** 1 Anesthesiology Clinic, Intensive Care Medicine, Emergency and Urgent Care, Anesthesiology Service, Centro Hospitalar Universitário de Santo António, Unidade Local de Saúde de Santo António, Porto, PRT

**Keywords:** airway management, difficult airway management, first-pass success, postoperative management, postoperative sore throat

## Abstract

Introduction

Postoperative sore throat (POST) is a frequent complication after general anesthesia with airway instrumentation. This study aimed to determine the incidence and severity of POST in patients admitted to the post-anesthesia care unit (PACU) of Centro Hospitalar Universitário de Santo António (CHUdSA), Porto, Portugal, to compare these findings with published data, and to identify perioperative variables associated with increased or decreased risk.

Methods

A retrospective analysis of 106 adult patients undergoing general anesthesia with airway instrumentation was performed. POST severity was assessed using the Visual Analog Scale (VAS). Demographic, anesthetic, airway management, and pharmacologic variables were collected and analyzed for associations with POST.

Results

POST occurred in 21.7% of patients, with 5% reporting moderate pain, all following endotracheal intubation. Difficult laryngoscopy and multiple intubation attempts were significantly associated with a higher incidence and severity of POST. No pharmacologic intervention demonstrated a protective effect.

Conclusions

Mechanical airway factors, particularly difficult laryngoscopy and repeated intubation attempts, were the strongest predictors of POST. Optimizing intubation technique and achieving first-pass success remain key strategies to reduce this complication, while pharmacologic measures showed no significant benefit.

## Introduction

Postoperative sore throat (POST) is a common and often underestimated complication following general anesthesia with airway instrumentation. Its incidence is reported to range from 33% to 61% [1-4], with a notable impact on patient satisfaction and overall perioperative experience. The pathophysiology of POST is primarily related to mechanical irritation of the airway mucosa, frequently associated with endotracheal intubation, though it can also occur with supraglottic airway devices (SADs).

The etiology of POST is complex and multifactorial, involving mucosal trauma, prolonged pressure on the tracheal wall, inflammation, and possibly reflux or chemical irritation [1,2]. Endotracheal intubation using stylet-loaded tubes, particularly with excessive curvature, has been shown to increase pressure on the anterior tracheal wall, leading to injury [1]. Additional procedural factors such as multiple intubation attempts, difficult laryngoscopy, or high cuff pressures have also been implicated [1,2,5].

Several risk factors have been consistently identified: female sex, younger age, longer duration of anesthesia, larger endotracheal tube (ETT) size, presence of naso/orogastric tubes, and double-lumen tubes [1-3,5]. While the use of videolaryngoscopy is associated with improved glottic visualization and higher first-pass success, its effect on POST remains inconclusive [1,6].

Pharmacological interventions aimed at preventing or mitigating POST include corticosteroids (e.g., dexamethasone), nonsteroidal anti-inflammatory drugs (NSAIDs) (e.g., ibuprofen), lidocaine (topical or IV), and N-methyl-D-aspartate (NMDA) receptor antagonists (e.g., ketamine, magnesium sulfate). While meta-analyses suggest some efficacy, particularly with topical agents and higher dexamethasone doses, results are heterogeneous and not always reproducible in routine practice [1,7-9].

This study aims to assess the incidence and severity of POST in the post-anesthesia care unit (PACU) of Centro Hospitalar Universitário de Santo António (CHUdSA), evaluate its alignment with previously reported rates, and identify perioperative variables associated with a higher or lower risk of POST in our institution.

## Materials and methods

This retrospective study was conducted at CHUdSA, a tertiary hospital in Porto, Portugal, providing a wide range of surgical specialties. All adult patients (≥18 years) undergoing surgery under general anesthesia with airway instrumentation between August and September 2024 were included. Exclusion criteria comprised head, neck, or upper airway surgery and recent or ongoing upper respiratory tract infection (within 30 days).

The primary outcome was the severity of POST, assessed using the Visual Analog Scale (VAS, 0-10), as originally described by Huskisson (1974) in The Lancet, a validated instrument for pain measurement [10]. Pain intensity scores were systematically recorded in the PACU by anesthesiologists, anesthesiology residents, or nurses as part of standard postoperative observations, and these data were subsequently extracted for analysis. Independent variables included demographic data (age, sex), American Society of Anesthesiologists (ASA) physical status, surgical specialty, airway device, number of intubation attempts, laryngoscopy grade, use of videolaryngoscope, stylet, or orogastric tube, and pharmacologic prophylaxis with lidocaine, dexamethasone, or ketamine.

For patients undergoing endotracheal intubation, we recorded the airway device, number of attempts, use of a stylet, and the laryngoscopy technique (direct vs. video-laryngoscopy), as documented in the anesthesia record. A total of 68 patients were intubated, of whom 56 underwent direct laryngoscopy, and 12 underwent video-laryngoscopy. No conversions from direct to video-laryngoscopy occurred. Routine lubrication of endotracheal tubes is not part of standard practice in our department and was not performed in this cohort. Lubrication is reserved for nasotracheal intubations, which were not included in the present analysis. A low threshold for first-attempt video-laryngoscopy was applied in the presence of predictors of a difficult airway. A stylet was used in 12 intubations (17.6%).

**Figure 1:**
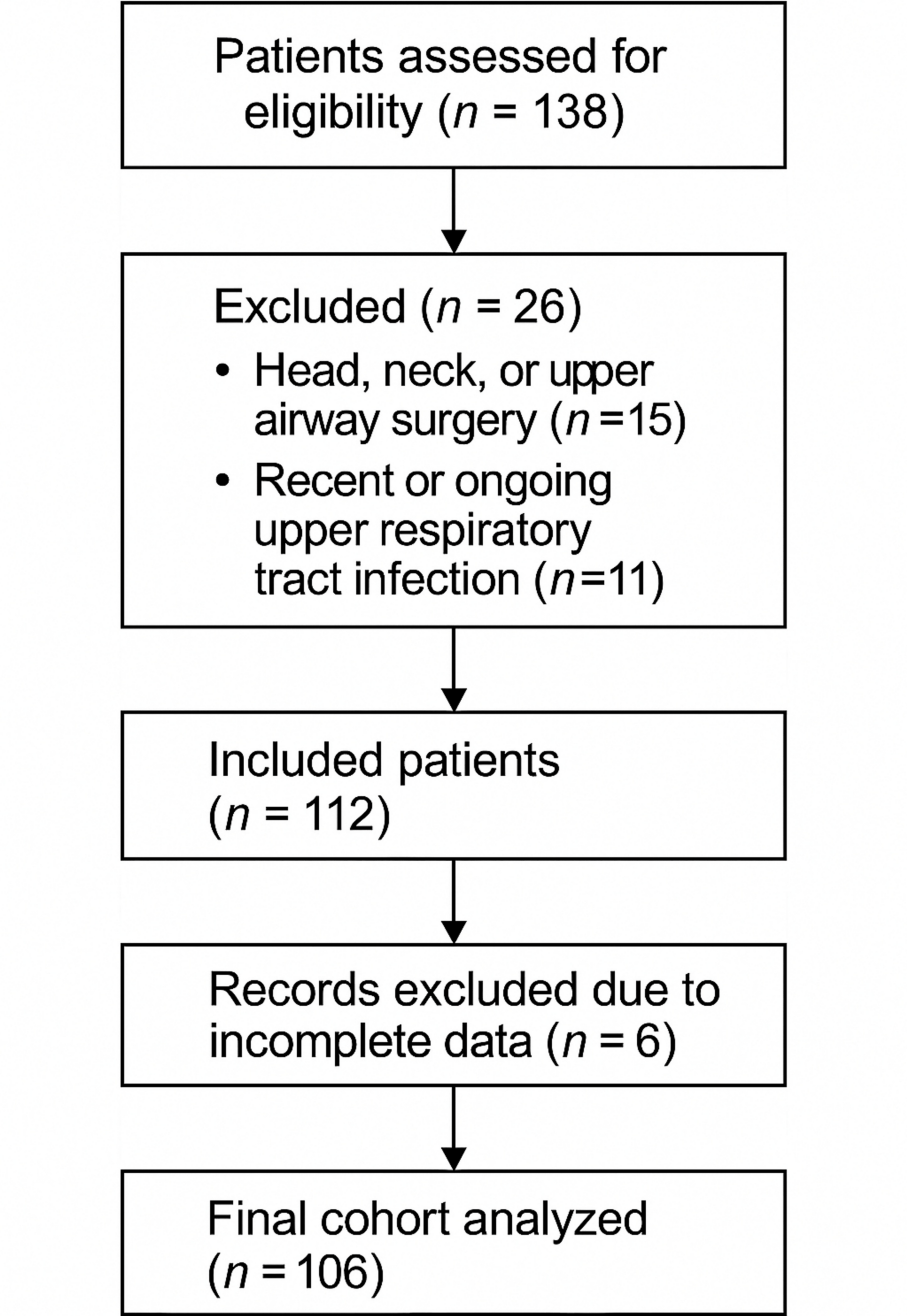
Cohort flowchart of patient inclusion and exclusion. The flowchart illustrates the patient selection process for the study. It shows the total number of patients assessed for eligibility (n = 138), those excluded due to head, neck, or upper airway surgery (n = 15) and respiratory infection (n = 11), and those with incomplete data (n = 6), resulting in a final sample of 106 patients included in the analysis.

All eligible patients during the study period were consecutively included. A total of 106 patients met the inclusion criteria. Data were collected retrospectively via anesthesia records and PACU observations (Figure 1).

Statistical analysis

Descriptive statistics were applied to summarize demographic and clinical data. Normality of continuous variables was assessed using the Shapiro-Wilk test and histogram analysis. Associations between POST severity (mild: VAS 1-3; moderate: VAS 4-7) and categorical variables were tested using the chi-square test, with a significance threshold of p < 0.05.

## Results

A total of 106 patients met the inclusion criteria. The mean age was 62 ± 15.3 years, and 70 (66%) were male (Table 1). Most participants were classified as ASA II-III (N = 73, 68.5%), and general surgery was the most frequent specialty (N = 38, 35.8%), followed by urology (N = 26, 24.5%), gynecology (N = 18, 17%), and orthopedics (N = 12, 11.3%) (Tables 2, 3).

**Table 1:** Sociodemographic characteristics of the study population. This table summarizes the demographic profile of the patients, including sex distribution and mean age, expressed as N (%) or mean ± standard deviation.

Variable	N (%) or Mean ± SD
Sex (Male)	70 (66%)
Sex (Female)	36 (34%)
Mean Age (years)	62 ± 15.3

**Table 2:** ASA physical status classification of the study population. This table presents the distribution of patients according to the American Society of Anesthesiologists (ASA) physical status classification. Most patients were classified as ASA III (41.5%), indicating a predominance of individuals with severe systemic disease.

ASA status	N (%)
ASA I	18 (17%)
ASA II	29 (27%)
ASA III	44 (41.5%)
ASA IV	15 (14%)

**Table 3:** Distribution of surgical specialties among included patients. This table lists the surgical specialties represented in the study population, showing the number and percentage of patients undergoing each type of procedure (N (%)).

Surgical specialty	N (%)
General surgery	38 (35.8%)
Urology	26 (24.5%)
Gynecology	18 (17%)
Orthopedics	12 (11.3%)
Other	12 (11.3%)

Of the 106 patients included, 68 (64.1%) underwent endotracheal intubation, and 38 (35.9%) were managed with a supraglottic airway device. Among the intubated patients, 56 (82.4%) were intubated with direct laryngoscopy and 12 (17.6%) with video-laryngoscopy. A stylet was used in 12 intubations (17.6%).

POST occurred in 23 (21.7%) patients. Among these, five (5%) patients reported moderate pain (VAS 4-7), all of whom had undergone endotracheal intubation (Table 4). Most patients (N = 100, 94.3%) required only one intubation attempt, and the majority of laryngoscopies were classified as easy (grade I). Difficult laryngoscopy was significantly associated with an increased incidence of POST (p = 0.001), and a higher number of airway management attempts associated with greater pain severity (p < 0.001) (Tables 4,5,6).

**Table 4:** Severity of postoperative sore throat (POST) according to the Visual Analog Scale (VAS). This table reports the distribution of postoperative sore throat severity based on VAS scores (0–10), expressed as N (%). It highlights the proportion of patients experiencing no, mild, or moderate symptoms.

POST (VAS)	0	1	2	3	4	5	6	7	8	9	10
N (%)	83 (78.3%)	2 (1.9%)	11 (10.4%)	5 (4.7%)	1 (0.9%)	1 (0.9%)	1 (0.9%)	2 (1.9%)	0 (0%)	0 (0%)	0 (0%)

**Table 5:** Association between laryngoscopy grade and postoperative sore throat (POST). This table shows the relationship between laryngoscopy grade (I–III) and the incidence of mild or moderate POST, including the corresponding p-value. Higher laryngoscopy grades were significantly associated with increased POST incidence. Mild POST includes POST with VAS between 1 and 3 and moderate between 4 and 7.

Severity of POST	Laryngoscopy grade			P- value
	Grade I	Grade II	Grade III	
Mild POST, N (%)	46 (75.4%)	10 (16.4%)	5 (8.2%)	0.001
Moderate POST, N (%)	0 (0%)	4 (80%)	1 (20%)	0.001

**Table 6:** Association between number of airway management attempts and postoperative sore throat (POST). This table presents the correlation between the number of intubation attempts and POST severity, expressed as N (%) with statistical significance (p < 0.001), showing that multiple attempts were associated with higher pain scores. Mild POST includes POST with VAS between 1 and 3 and moderate between 4 and 7.

	Number of airway management attempts			P- value
	1	2	3	
Mild POST, N (%)	99 (98%)	2 (2%)	0 (0%)	<0.001
Moderate POST, N (%)	3 (60%)	1 (20%)	1 (20%)	<0.001

The use of a stylet was not associated with a higher incidence of postoperative odynophagia (p = 0.038), nor was the use of an oro/nasogastric tube (p = 0.552). Similarly, videolaryngoscopy did not show a protective effect compared with direct laryngoscopy (p = 0.145). The use of supraglottic airway devices was also not associated with a reduction in POST incidence when compared with orotracheal intubation (p = 0.087).

Regarding pharmacologic interventions, none of the evaluated agents, i.e., dexamethasone, lidocaine, ketamine, paracetamol, tramadol, NSAIDs, morphine, metamizole, magnesium sulfate, or dexmedetomidine, demonstrated a statistically significant protective effect against the development of POST.

## Discussion

The overall incidence of POST observed in this study (21.7%) lies at the lower end of the range reported in the literature (30-60%) [1-4]. This finding suggests that our institution's consistent adherence to airway management protocols and careful technique may reduce airway-related morbidity. The statistically significant association between difficult laryngoscopy and higher POST incidence (p = 0.001), as well as between multiple intubation attempts and greater symptom severity (p < 0.001), underscores the importance of mechanical factors in the development of POST. Similar associations have been reported in recent studies highlighting traumatic or repeated intubation as key predictors of sore throat following general anesthesia [5,11].

Regarding the use of videolaryngoscopy and stylets, our findings did not demonstrate a protective effect against POST (p = 0.145 and p = 0.038, respectively). These results align with recent evidence suggesting that technological advances do not, by themselves, reduce POST incidence unless accompanied by improved first-pass success and operator experience. Quality-improvement programs focusing on first-attempt success have been shown to lower airway trauma and postoperative complications [2,6].

In our institution, video laryngoscopy is preferentially used in patients with predicted difficult airway characteristics, who inherently carry a higher risk of mucosal trauma. This indication bias may partly explain why video-laryngoscopy did not demonstrate a protective effect against POST in this cohort.

On the pharmacologic side, none of the agents evaluated in our study, including dexamethasone, lidocaine, ketamine, paracetamol, tramadol, NSAIDs, morphine, metamizole, magnesium sulfate, or dexmedetomidine, showed a statistically significant protective effect. This is consistent with the most recent meta-analyses, which report considerable heterogeneity across pharmacologic prophylaxis studies for POST and an overall lack of definitive evidence supporting a single drug or dosing regimen [7-9,12,13]. For instance, despite isolated positive findings for topical corticosteroids or lidocaine preparations, the global evidence remains inconclusive, and pharmacologic interventions cannot substitute for careful airway manipulation [7,8,12,13].

The contrast between the evident predictive value of mechanical airway factors and the inconsistency of pharmacologic prophylaxis highlights a practical implication: optimizing intubation technique should remain the cornerstone of POST prevention, while pharmacologic measures may serve as adjuncts [1,6,14] This message aligns with current clinical trends emphasizing atraumatic airway management, first-pass intubation, and cuff pressure monitoring as essential steps to minimize postoperative airway symptoms [4,7,12]. Beyond symptom prevention, these strategies may also improve patient satisfaction and recovery time, as supported by broader perioperative quality literature [3,10].

The study has limitations inherent to its retrospective design, including potential documentation bias and a relatively small number of patients reporting moderate POST (n = 5), which limits the statistical power for subgroup analysis. Future prospective multicenter studies with standardized reporting of airway variables (such as cuff pressure, number of attempts, and device type) are warranted. In addition, new randomized controlled trials focusing on pharmacologic prophylaxis with uniform designs could clarify the role of specific agents. For example, recent meta-analyses of preoperative ketamine gargles have demonstrated a significant reduction in POST severity and incidence (Relative-Risk - RR ≈ 0.48) [15].

## Conclusions

In this study, difficult laryngoscopy and multiple intubation attempts emerged as the most significant predictors of POST, underscoring the key role of mechanical airway factors in its development. None of the pharmacologic interventions evaluated demonstrated a significant protective effect, highlighting the current inconsistency of drug-based prophylaxis in this setting. These findings reinforce that minimizing airway trauma remains the most reliable and effective strategy to prevent POST. A multimodal, but technique-centered, approach should therefore be prioritized, focusing on gentle laryngoscopy, careful tube handling, and maximizing first-pass intubation success. Clinicians are encouraged to consider non-pharmacologic strategies as the foundation of POST prevention, reserving pharmacologic agents as complementary measures. Future studies with larger cohorts and prospective designs are warranted to further clarify the role and comparative efficacy of pharmacologic interventions in mitigating postoperative airway discomfort.
